# Visual feedback using electrical impedance tomography during inhalation therapy for chronic obstructive lung disease: a pilot study

**DOI:** 10.1038/s41598-026-67510-0

**Published:** 2026-08-24

**Authors:** Svenja Stolz, Rosa Meyer, Torben Rixecker, Robert Bals, Philipp M. Lepper, André Becker

**Affiliations:** 1 Dept. of Internal Medicine V – Pneumology, Allergology and Critical Care Medicine, University Hospital of Saarland and Saarland University, Kirrberger Str. 1, 66421 Homburg, Saar Germany; 2 Dept. of Internal Medicine, Pneumology and Critical Care Medicine, University Hospital OWL of the University of Bielefeld, Bielefeld, Germany

**Keywords:** COPD, Electrical impedance tomography, Visual feedback, Inhalation, Diseases, Health care, Medical research, Physiology

## Abstract

Airflow limitation and hyperinflation contribute to increased respiratory workload and dyspnea in COPD. Inhalation therapy plays a crucial role, but its effectiveness depends on correct technique. Electrical impedance tomography (EIT) offers real-time visualization of ventilation and may improve inhalation technique, when used as visual feedback. This study aimed to investigate the impact of feedback-guided inhalation using EIT on lung function, specifically targeting obstructive and hyperinflation parameters. In this prospective, randomized, single-blinded study, 29 patients with COPD were divided into a control group (no feedback) and an intervention group (inhalation using visual feedback). Body plethysmography was performed before and after a single inhalation session. The primary outcome was the change in lung function parameters. Secondary outcomes included real-time EIT data during inhalation and a follow-up questionnaire assessing subjective benefit. No differences were observed in obstructive lung parameters (FEV1) between groups, but the intervention group showed a significant FRC reduction compared to the control group (control − 0.1 L vs. intervention − 0.4 L, p < 0.05). Resistance indices (sRtot/sReff) tended to increase in the intervention group whereas a decrease was noted in control. No in between differences in EIT-derived data or subjective benefit at follow-up. Visual feedback using lung EIT may improve inhalation technique and breathing mechanics, which may contribute to reduced hyperinflation. However, changes in breathing pattern may also explain changes in lung parameters, highlighting the complex lung volume, ventilation and airway resistance interplay. Future research should investigate repeated EIT-feedback effects on lung function and disease symptoms.

*Trial registration***:** Registered at: DRKS.de; Clinical Trial Registration Number: DRKS-IDDRKS00037626; Registration Date: 11.08.2025.

**Supplementary Information:**

The online version contains supplementary material available at 10.1038/s41598-026-67510-0.

## Introduction

Chronic obstructive pulmonary disease (COPD) is a major global health challenge, contributing significantly to morbidity and mortality^1–3^. Although COPD has multiple causes, the main driver is airway damage from inhaled noxious substances. Effects include chronic inflammation and airflow limitation, presenting as incompletely reversible obstruction with chronic bronchitis. Altered respiratory mechanics due to air-trapping, hyperinflation and finally the development of emphysema can contribute to a further deterioration in ventilation.

Therapeutic drug approaches, primarily inhaled, are intended to reduce obstruction and decrease inflammation by inhalation of bronchodilators and corticosteroids^4,5^. Effective inhalation therapy requires sufficient drug deposition in the lungs. In COPD diaphragm dysfunction and hyperinflation impair ventilation and reduce inspiratory flow—a key factor for drug delivery^6^. Incorrect inhalation technique often limits treatment success^7,8^. Feedback training can improve technique, adherence and outcome^9^. Although training can improve inhalation technique, it is not clear which method is most effective in identifying and correcting inhalation errors^10^. Visually supported feedback was found to be beneficial for patients with COPD in rehabilitation^11,12^.

Electrical lung impedance tomography (EIT) is a bedside method visualizing real-time ventilation via thoracic impedance changes during ventilation. Alveolar air changes during ventilation alter lung impedance, enabling real-time 2D imaging of chest sections to show regional ventilation, the distribution of air and parameters of regional ventilation^13,14^. Lung EIT is primarily applied in ventilated patients, such as in ARDS (Acute Respiratory Distress Syndrome) or during weaning^15^. Lung EIT has not yet been used as feedback to visualize or improve inhalation in patients with COPD. This study investigated EIT as visual feedback for potential benefits of real-time visualized ventilation in inhalation therapy.

## Material and methods

### Study design and population

This prospective randomized, single-blinded study was performed in the Department of Internal Medicine V, Pneumology, Allergology and Critical Care Medicine of the University Hospital of Saarland and Saarland University, Homburg/Saar, Germany. Adults diagnosed with COPD who were on continuous inhalation therapy and in a stable disease stage, i.e. no sudden worsening of cough, sputum production, shortness of breath or recent hospitalization were included in this study. The protocol was approved by the local ethics review committee (No. 87/23). Written informed consent was obtained from the participants. Exclusion criteria included contraindications for EIT as specified by the manufacturer (pacemakers or ICDs (implantable cardioverter-defibrillators), BMI > 50, unstable thorax including open chest injuries, large burns or wounds on the thorax), signs of acute exacerbation of COPD or cognitive impairments and language barriers.

### Study protocol

Participants with a history of COPD were recruited during routine pulmonary clinic visits, consented, randomized into control or intervention group, and underwent body plethysmography before the study. Static and dynamic lung volumes were measured using a whole-body plethysmograph (Vyntus™ BODY, Vyaire Medical, Höchberg, Germany) by trained pulmonary function technicians according to ATS/ERS recommendations. The device underwent routine daily calibration and quality control according to the manufacturer’s instructions. Before the intervention, all participants received standardized training in the correct use of the pressurized metered-dose inhaler to minimize handling errors. Participants were instructed to shake the inhaler vigorously, exhale slowly before inhalation, slightly extend the head, actuate the inhaler at the beginning of inspiration, inhale slowly and deeply towards maximal inspiration, and hold their breath for 5–10 s before exhaling slowly. Breath-hold duration was standardized using a timer operated by the investigator. All participants were connected to an EIT device (PulmoVista® 500, Dräger, Germany) via a chest belt placed at the 4th–6th intercostal space. Electrode positioning was performed according to the international TREND consensus recommendations^14^. In the control group, only the examiner saw the EIT monitor, whereas in the intervention group both examiner and participants observed real-time ventilation at the EIT monitor. The control group was told that the examiner would observe their breathing in real-time. Participants allocated to the intervention group additionally received real-time visual feedback from the EIT monitor. During an initial baseline recording, the basic principle of EIT was explained in simplified terms, emphasizing that the displayed colour changes represented regional lung ventilation. Using a standardized instruction protocol, participants were encouraged to achieve the largest possible coloured ventilation area while maximizing the brightness of the displayed ventilation signal during inspiration, maintain the image during the breath-hold phase, and observe the gradual changes during expiration. These visual cues were intended to promote deep inhalation and breath-hold rather than to optimize specific EIT-derived indices. Both groups performed 10 resting breathing cycles, then inhaled 100 µg salbutamol (pressurised inhaler) with a minimum 5-s to a maximum 10 s breath hold. The procedure was repeated four times within 5 min. After inhalation, body plethysmography was repeated approximately 5–15 min after completion of the inhalation procedure, in accordance with the study protocol. This timing was selected to remain consistent with the GOLD recommendations^4^ for post-bronchodilator spirometry and to capture the immediate physiological effects of the visual-feedback intervention. During the experiment, real-time data of EIT during inhalation were recorded for further analysis. Study protocol, see supplementary Fig. 1.

### Measurements and data recording

Static and dynamic lung volumes were measured by using body plethysmography (FEV1, FEV1/FVC, FVC, TLC, RV, FRC, sRtot, sReff) under supervision by trained medical-technical assistants. Pre- and post-inhalation plethysmography values were recorded and compared between groups. Differences in lung function before and after the experiment were calculated for each patient separately before calculating and comparing group values. Baseline labs and CAT (COPD Assessment Test) scores were recorded. After three months, follow-up included a repeated CAT score and an ordinal questionnaire on subjective feedback benefit. Recorded EIT data was sampled at 30 Hz, transferred to an external computer and analyzed using dedicated software (PulmoVista SW 1.30 and EITDiag V1.7), both supplied by Dräger. The following EIT-derived indices were calculated:

### Global tidal volume image (TID)

TID was defined as the global tidal impedance variation within the predefined lung region of interest (ROI), as provided by the PulmoVista 500 software. Because EIT measures relative impedance changes rather than absolute lung volumes, TID values obtained during inhalation were expressed relative to baseline tidal breathing. TID was analysed during 3–5 resting breathing cycles before inhalation (baseline) and during medication inhalation. Relative changes in TID between baseline breathing and inhalation were compared between the control and the intervention groups.

### Global inhomogeneity index (GI)

**Fig. 1:**
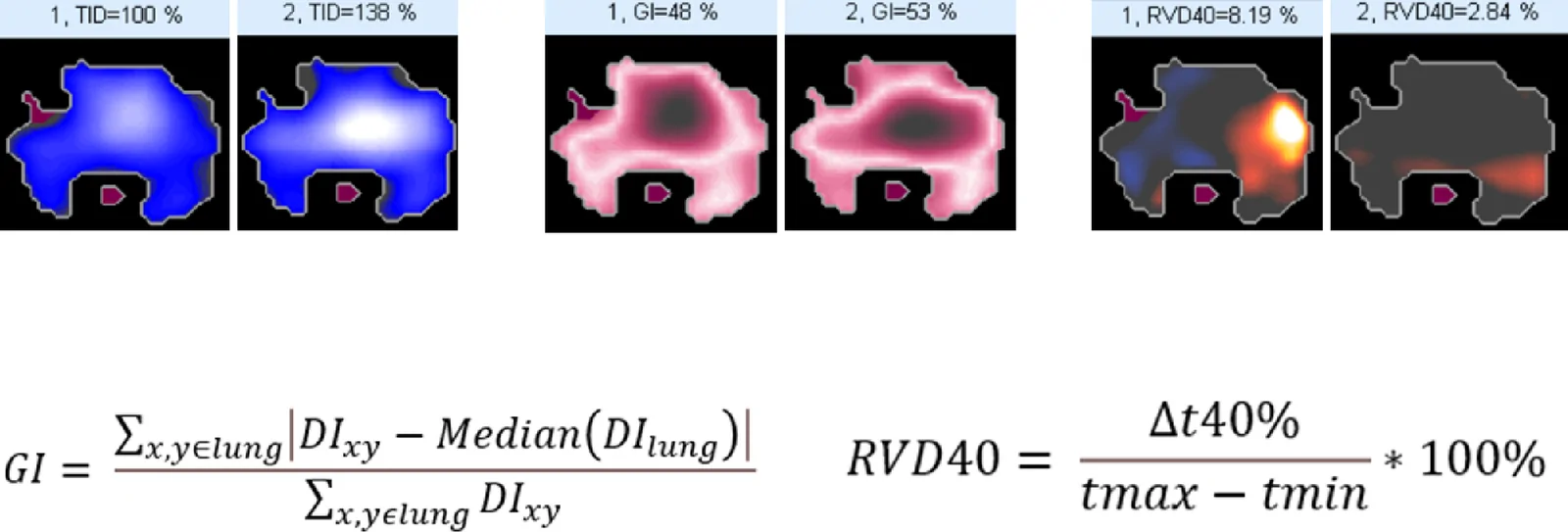
Top: Left: TID, Tidal impedance difference; Middle: GI, Global inhomogeneity index; Right: RVD, Regional ventilation delay index; Examples of a patient from the intervention group during normal breathing cycles (1) and during inhalation (2). TID values during inhalation are expressed relative to the normal breathing cycle (set at 100%). Global tidal volume image (TID) is color-coded from dark blue (low ventilation) to white (high ventilation). In this example, difference between tidal breathing and inhalation is 38%. GI is color-coded from dark red (homogeneous) to bright red (inhomogeneous). In this example GI increases from 48% before inhalation to 53% (indicating less homogeneous ventilation) during inhalation. Regional ventilation delay (RVD40), color-coded in orange, shows a reduction in ventilation delay from 8.19% to 2.84% during inhalation. Bottom: GI: Global inhomogeneity index; DI: Value of the differential impedance in the tidal images; DI_xy_: Pixel in the identified lung area; DI_lung_: Total pixels representing the lung area. RVD40: Regional ventilation delay inhomogeneity; time difference between initiation of inspiration and 40% ventilation achieved, t_max_–t_min_ = Inspiration time

The GI is derived from a global tidal volume image. GI is calculated as the difference between the median value of all pixels within a tidal volume image and the corresponding value of each individual pixel^16^. The GI reflects the variability of ventilation distribution. Similar to TID, GI was analysed before inhalation (baseline) and during inhalation (changes in GI) and compared between control and intervention group. A lower GI indicates a more homogeneous distribution of ventilation, while a higher GI index suggests a more inhomogeneous ventilation. Calculation of GI is displayed in Fig. 1.

### Regional ventilation delay inhomogeneity (RVDI)

Regional ventilation delay (RVD) was defined as the time interval between the onset of inspiration and the time point at which 40% of the maximal regional impedance change during tidal inspiration was reached^17,18^. RVD was calculated for each pixel, and RVDI was defined as the standard deviation of RVD across all lung pixels, reflecting spatial inhomogeneity of regional filling. Lower RVDI values indicate more homogeneous and synchronous ventilation, whereas higher values reflect increased regional delay and inhomogeneity. Differences between resting breathing and the inhalation were analysed to assess changes in regional ventilation timing during inhalation. Calculation of RVDI is displayed in Fig. 1.

### Statistical analysis

Statistical analysis was performed using IBM SPSS Statistics (version 29.0.1.0.) Figures in the results section were generated with Python (version 3.12.4.) and randomization was conducted using R (version 4.2.2.) Normal distribution was assessed with Shapiro–Wilk test. As data were not normally distributed, between-group comparison of unpaired continuous variables was performed using the Mann–Whitney test, and categorical variables from follow-up surveys were analysed using the chi-square test. Results are reported as control versus intervention. Due to non-normal distribution data is presented as median (25th–75th percentile), unless stated otherwise. Lung function results are reported in liters (L) and as height-adjusted percent of predicted reference values (% pred.). Predicted reference values are based on a healthy reference population^18,19^. The response of lung function parameters was determined for each individual patient by calculating the difference between post- and pre-inhalation values. These derived variables were then used for further statistical analysis between groups, in this case using a Mann–Whitney test. P-value < 0.05 was considered statistically significant.

## Results

### General

A total of 30 participants were recruited; 29 were analyzed (n = 15 control group vs. n = 14 intervention group). One control participant was excluded due to incomplete plethysmography. The participant declined the repeated body plethysmography examination of their own free will. Intervention or intervention group refers to EIT-feedback.

### Baseline demographics and laboratory findings

The study population comprised 29 patients, including 15 female and 14 male participants, with an age of 64 years vs. 65.5 years (p > 0.05) and a BMI of 27 kg/m^2^ vs. 24.3 kg/m^2^ (p > 0.05), and Pack-years of 40 vs. 40 (p > 0.05). The median CAT score at inclusion was 26 / 40 points vs. 24.3 / 40 points with no significant difference between groups (p > 0.05). Spirometric GOLD classification (FEV_1_) was comparable between groups (Table 1). Inflammation markers, including leukocytes, C-reactive protein and eosinophils, were within normal range based on median values and showed no significant group differences, except for C-reactive protein (which was within normal range). The basic laboratory values are listed in Table 1.

**Table 1:** Baseline characteristics and laboratory findings of COPD patients in the control group (n = 15) compared to the intervention group (n = 14).

	Control; N = 15 median (Q1–Q3)	Intervention; N = 14 median (Q1–Q3)	p-value
Age (years)	64 (60.5–76)	65.5 (60.3–69.5)	0.847
Sex (male, N; %)	8/15; 53.3%	6/14; 42.9%	0.853
Body mass index (kg/m^2^)	27 (26.2–33.1)	24.3 (21.3–32.8)	0.425
Pack Years	40 (30–50)	40 (18.8–50)	0.533
CAT score	26 (12.8–29)	24.5 (21.3–30.8)	0.949
GOLD grade (N)			0.400
I	3	0	
II	2	3	
III	6	7	
IV	4	4	
Leukocytes (g/l)	9.0 (8–11.3)	8.2 (6.5–11)	0.339
CRP (mg/dl)	4.4 (1.5–8.6)	1.2 (0.6–2.2)	**0.046**
Eosinophils (/µl)	114.3 (83.1–345) **N = 13/15**	115 (69.1–185.8) **N = 12/14**	0.270

CAT: COPD assessment test; GOLD: Global Initiative for Chronic Obstructive Lung Disease; Hb: Haemoglobin; CRP: C-reactive protein. Bold values indicate statistical significance (p < 0.05).

### Body plethysmography results control group vs. intervention group

Lung function results before the experiment (inhalation) are listed in Table 2.

**Table 2:** Pulmonary function before inhalation in control group (n = 15) and intervention group (n = 14). Absolute measurements are supplemented by values normalized to the expected values of a healthy population (%).

	Control; N = 15 median (Q1–Q3)	Intervention; N = 14 median (Q1–Q3)	p-value
FEV_1_ (L)	1.2 (0.6–1.7)	0.9 (0.7–1.2)	0.451
FEV_1_ (%)	42 (22–65)	33.5 (27–42)	0.451
FEV_1_/FVC	50.7 (43.1–66.9)	49.6 (38.5–73)	0.914
PEF (L)	3.1 (2.2–4.7)	3.0 (1.7–3.6)	0.451
PEF (%)	44 (33–72)	44.5 (29–52.5)	0.377
FVC (L)	2.0 (1.7–2.8)	1.7 (1.5–2.1)	0.252
FVC (%)	58 (48–77)	52 (47–61)	0.172
TLC (L)	6.7 (6.2–8.1)	6.5 (5.5–8.9)	0.983
TLC (%)	123 (107–139)	126.5 (93–141)	0.949
RV (L)	4.8 (3–5)	4.7 (3.6–6.5)	0.621
RV (%)	208 (144–242)	228.5 (146–253)	0.533
FRC (L)	5.0 (3.5–5.8)	5.3 (4–7.5)	0.621
FRC (%)	176 (123–195)	189.5 (118–209)	0.533
sRtot (kPa*s)	5.3 (1.6–8.1)	4.0 (2–7.8)	0.914
sRtot (%)	448 (161–840)	384.5 (167–747)	0.847
sReff (kPa*s)	4.7 (1.5–5.8)	3.3 (1.7–7.2)	0.780
sReff (%)	400 (154–606)	310 (153–644)	0.914

FEV_1_: Forced Expiratory Volume in one second; FEV_1_/FVC: Ratio of Forced Expiratory Volume in one second to Forced Vital Capacity; PEF: Peak Expiratory Flow; FVC: Forced Vital Capacity; TLC: Total Lung Capacity; RV: Residual Volume; FRC: Functional Residual Capacity; sRtot: Specific Total Airway Resistance; sReff: Specific Effective Airway Resistance;

Results after the experiment (inhalation) are listed in Table 3.

**Table 3:** Pulmonary function after inhalation in control group (n = 15) and intervention group (n = 14).

	Control; N = 15 median (Q1–Q3)	Intervention; N = 14 median (Q1–Q3)	p-value
FEV1 (L)	1.2 (0.8–1.7)	1 (0.7–1.2)	0.377
FEV1 (%)	40 (28–65)	34.5 (28–48)	0.354
FEV1/FVC	54 (43.1–68.6)	46.5 (37.7–65.7)	0.505
PEF (L)	3.3 (2.1–4.8)	2.7 (2.2–3.8)	0.505
PEF (%)	41 (28–74)	43 (35–51)	0.683
FVC (L)	2.0 (1.6–3.1)	1.9 (1.6–2.2)	0.533
FVC (%)	54 (51–83)	53 (50–66)	0.400
TLC (L)	7.1 (6.4–7.9)	6.6 (5–8.3)	0.561
TLC (%)	123 (104–142)	115 (91–136)	0.451
RV (L)	4.5 (2.8–5.2)	4.3 (2.8–5.5)	0.949
RV (%)	199 (137–271)	196.5 (127–244)	0.747
FRCpl (L)	5.5 (3.4–5.9)	5.2 (3.6–7)	0.652
FRCpl (%)	173 (119–215)	168 (106–207)	0.983
sRtot (kPa*s)	3.9 (1.5–5.9)	4.2 (2.1–7.1)	0.621
sRtot (%)	332 (160–617)	402 (193–685)	0.652
sReff (kPa*s)	3.8 (1.5–5.6)	3.7 (1.8–6.1)	0.652
sReff (%)	325 (152–580)	349 (166–539)	0.715

Absolute measurements are supplemented by values normalized to the expected values of a healthy population (%).

FEV1: Forced Expiratory Volume in one second; FEV1/FVC: Ratio of Forced Expiratory Volume in one second to Forced Vital Capacity; PEF: Peak Expiratory Flow; FVC: Forced Vital Capacity; TLC: Total Lung Capacity; RV: Residual Volume; FRC: Functional Residual Capacity; sRtot: Specific Total Airway Resistance; sReff: Specific Effective Airway Resistance.

Calculated differences, before-after inhalation compared between groups are displayed in Table 4.

**Table 4:** Calculated differences in pulmonary function before and after inhalation in control group (n = 15) and intervention group (n = 14).

	Control; N = 15 median (Q1–Q3)	Intervention; N = 14 median (Q1–Q3)	p-value
FEV1 (L)	0.1 (0.0 to 0.2)	0.0 (0.0 to 0.1)	0.425
FEV1 (%)	3 (0.0 to 5)	0.5 (− 1 to 4)	0.468
FEV1/FVC	2.1 (− 3.8 to 4.6)	- 1.3 (− 4.6 to 3)	0.377
PEF (L)	0.1 (− 0.3 to 0.2)	0.0 (− 0.3 to 0.5)	0.628
PEF (%)	− 1 (− 4 to 3)	0 (− 4.5 to 8.5)	0.722
FVC (L)	0.1 (− 0.1 to 0.2)	0.1 (− 0.1 to 0.3)	0.511
FVC (%)	2 (− 3 to 6)	2 (− 2.3 to 9)	0.456
TLC (L)	− 0.1 (− 0.3 to 0.5)	− 0.4 (− 0.7 to 0.1)	0.149
TLC (%)	0.0 (− 7 to 10)	− 5 (− 13.5 to 1.8)	0.204
RV (L)	− 0.2 (− 0.5 to 0.4)	− 0.5 (− 1.1 to 0.1)	0.230
RV (%)	− 7 (− 25 to 20)	− 19 (− 42.8 to 5.3)	0.213
FRC (L)	− 0.1 (− 0.2 to 0.4)	− 0.4 (− 0.7 to − 0.1)	**0.040**
FRC (%)	− 2 (− 6 to 12)	− 11 (− 22.3 to − 2.8)	**0.031**
sRtot (kPa*s)	− 0.6 (− 1.3 to − 0.2)	− 0.1 (− 0.3 to 0.2)	**0.020**
sRtot (%)	− 50 (− 113 to − 18)	− 8.5 (− 29.3 to 17.8)	**0.033**
sReff (kPa*s)	− 0.5 (− 1.1 to − 0.1)	0.01 (− 0.4 to 0.2)	**0.033**
sReff (%)	− 43 (− 106 to − 7)	1 (− 32.5 to 20.3)	**0.046**

Group-level statistics were calculated from individual pre-post difference values.

FEV1: Forced Expiratory Volume in one second; FEV1/FVC: Ratio of Forced Expiratory Volume in one second to Forced Vital Capacity; PEF: Peak Expiratory Flow; FVC: Forced Vital Capacity; TLC: Total Lung Capacity; RV: Residual Volume; FRC: Functional Residual Capacity; sRtot: Specific Total Airway Resistance; sReff: Specific Effective Airway Resistance. Bold values indicate statistical significance (p < 0.05).

**Fig. 2:**
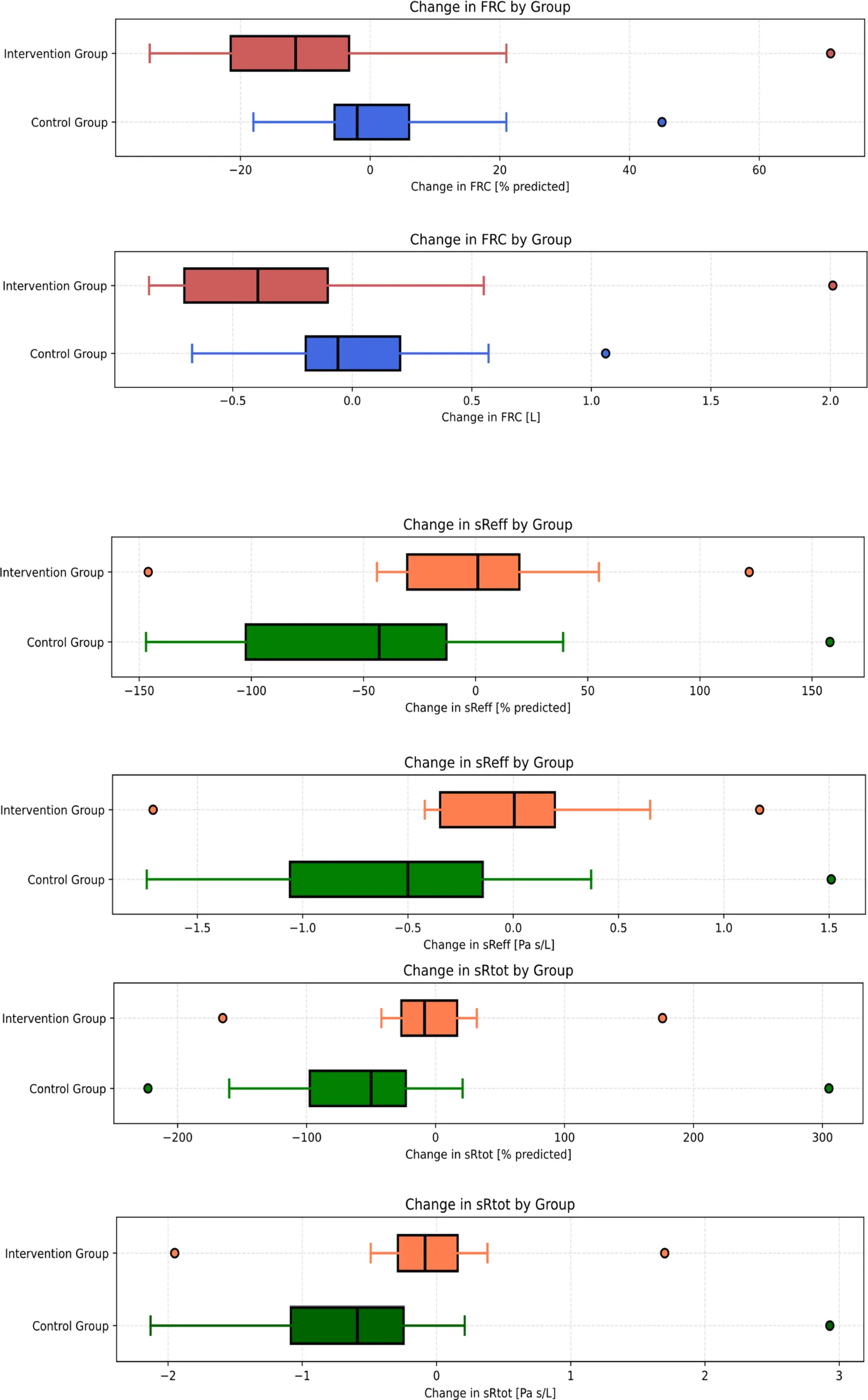
Top: Change in FRC (% predicted) between groups after inhalation and change in FRC (L) between groups after inhalation. Middle: Change in sR_eff_ (% predicted) between groups after inhalation and change in sR_eff_ (Pa*s/L) between groups after inhalation. Bottom: Change in sR_tot_ (% predicted) between groups after inhalation and change in sR_tot_ (Pa*s/L) between groups after inhalation.

Prior to the intervention, there was no difference between the groups, respectively in terms of FEV1 (1.2 L vs. 0.9 L, p > 0.05) and FVC (2 L vs. 1.7 L, p > 0.05). Total lung capacity (TLC) showed similar values in both groups before the intervention (6.7 L vs 6.5 L, p > 0.05), which exceeded 100% of the predicted TLC in both groups (123% pred. vs. 126.5% pred.). The residual volume (RV) was pathologically elevated in both groups (4.8 L, 208% pred. vs. 4.7 L, 228% pred., p > 0.05) indicating emphysema. Likewise, functional residual capacity (FRC) was increased indicating hyperinflation in both groups (5.0 L, 176% pred. vs. 5.3 L, 189% pred., p > 0.05). After the intervention, both groups showed a similar median increase in FVC (+ 0.1 L, + 2% pred. control vs. intervention, p > 0.05). Regarding FEV1, the control group exhibited a slight median increase while the intervention group showed no relevant change (+ 0.1 L, + 3% pred. vs. ± 0.0 L, + 0.5% pred., p > 0.05). A reduction in TLC (− 0.1 L, 0.0% pred. vs. − 0.4 L, − 0.4% pred.) and RV (− 0.2 L, − 7% pred. vs. − 0.5 L, − 19% pred.) was observed, more in the intervention group than in the control group, without reaching statistical significance (p > 0.05). Notably, FRC showed a significant improvement in the intervention group. A reduction in FRC of − 0.4 L (− 11% pred.) was observed, compared to a reduction of − 0.1 L (− 2% pred.) in the control group, reaching a significant difference (p < 0.05). Of note, the parameters for specific airway resistance (sRtot, sReff) were significantly lower in the control group compared to the intervention group. Total airway resistance decreased by a median of 50% pred. in the control group versus 8.5% pred. in the intervention group (p < 0.05). Similarly, the sReff decreased by − 43% pred. in the control group compared to an increase of + 1% pred. in the intervention group (p < 0.05). Significant differences are shown in Fig. 2.

### EIT-derived parameters TID, GI and RVDI

EIT-derived parameters included three key parameters: TID, GI, and RVDI. In the control group, data from 15 patients were evaluable for analysis, whereas in the intervention group, EIT data were evaluable for only 11 patients because of artefacts during recording. TID showed no significant group difference during inhalation (+ 237 vs. + 277, p > 0.05). GI showed decreased values in both groups indicating more homogeneity during inhalation in both groups. The control group had a median GI of 48.9 pre-inhalation vs. 42.81 (− 4) during inhalation (p > 0.05), the intervention group showed a median decrease in GI from 53.9 pre-inhalation to 45.3 (− 8.3) during-inhalation, level of significance was not reached (p > 0.05). RVDI differed slightly between groups. While the control group presented a RVDI with a median of 7.4 before and 7.6 during inhalation, the intervention group demonstrated a RVDI with median of 8.1 before and higher RVDI of 11.5 during inhalation (p = 0.071) indicating more delay during inhalation. The median difference of RVDI between pre- and inhalation was 0.2 vs. − 0.2 (p > 0.05). Parameters are shown in supplementary Table 5, example of EIT data is visualised in Fig. 1.

### Follow-up CAT-score and subjective assessment of participants

Follow-up interview was completed in 26 of 29 enrolled patients. Two patients passed away during the follow-up period, one withdrew consent for a phone interview. 12 /14 patients in the intervention group and 14 / 15 patients in the control group completed the follow-up. Patients without follow-up interview were excluded from CAT-analysis. The median time between study participation and follow-up was 4.8 months. At follow-up, the median CAT score across all participants was 23.5 points, representing a median reduction of 1.9 points compared to baseline. In the control group, the CAT score decreased by a median of 2.4 points, whereas in the intervention group, the median reduction was 1.0 points, see suppl. Table 5. Supplementary Table 6 summarizes quantitative results in the intervention group, values are ordered from 1 = strongly disagree, to 5 = strongly agree. The Results indicate that the EIT image was generally perceived as helpful (Question 1; median: 4). Additionally, most patients found the EIT image to be understandable (Question 2; median: 4.5), with no participants rating their understanding as "disagree" or "strongly disagree." The guidance and feedback were rated as highly helpful (Question 4; median = 5). No patient rated the guidance or feedback below "3 = neutral." No clear trend was seen in perceived inhalation effectiveness (Question 5; median: 3.0). During the follow-up, all participants were asked whether they perceived any benefit from participating in the study. Responses were categorized into three groups: "No benefit or new insights," "Subjective benefit from the EIT intervention," and "Subjective benefit from inhalation training." None of the participants in the control group reported deriving any benefit from the study. In the intervention group, the responses of three patients fell into the category "No benefit or new insights." Four patients reported benefiting from inhalation training but not from visual feedback. Five patients described a subjective benefit specifically attributed to the EIT-feedback.

## Discussion

In this randomized pilot study, visual feedback using EIT during inhalation therapy was associated with a greater reduction in FRC than standard inhalation training. No between-group differences were observed for FEV1, FVC, EIT-derived ventilation parameters, or CAT scores. While several participants reported subjective benefits from visual feedback, these findings remained exploratory. In contrast, specific airway resistance improved significantly in the control group. Overall, these results suggest that EIT-guided visual feedback may influence hyperinflation without producing measurable short-term changes in conventional spirometric or regional ventilation parameters.

Interpreting the main finding, FRC reduction, requires considering static and dynamic hyperinflation, which is mainly induced by destruction of the lung parenchyma with diminished inward-directed elastic fibers. This results in a pressure–volume curve shifted upward and to the left^19^. Clinically, hyperinflation increases inspiratory workload, impairs gas exchange and alters cardiopulmonary interaction contributing to dyspnea and reduced performance^19,20^. Previous studies show that bronchodilation response does not always correlate with expiratory flow measure. Symptomatic improvement, such as reduced dyspnea often aligns with decreased FRC, rather than increased FEV1^21^. Importantly, the absence of FEV1 changes does not always imply a lack of bronchodilator effect. In COPD, acute improvements in expiratory flow are often small, while changes in hyperinflation more accurately reflect physiological benefit. Consequently, FRC might represent a more sensitive parameter than FEV1 to detect short-term physiological effects of improved inhalation technique in COPD. In our experiment, we were unable to detect any changes in FEV1; however, a significant change in FRC was observed in the intervention group, with a decrease of 0.4 L in median (- 11% pred). In the literature, an increase in FRC is generally considered clinically relevant when it exceeds approximately 120% of predicted value, corresponding to the upper limit of normal in a healthy population^22–24^. However, several reports suggest that a reduction in dynamic hyperinflation is associated with clinically meaningful improvements in exercise capacity and reduction in dyspnea symptoms^19,25–27^. The observed reduction in FRC in the intervention group may reflect more effective inhalation facilitated by real-time feedback, which may have contributed to the observed reduction in FRC. Alternatively, this reduction may indicate improved breathing mechanics, particularly during exhalation, mediated by feedback-guided respiratory control. In the future, this effect could potentially be enhanced through structured respiratory training incorporating visual feedback. As demonstrated in previous studies in context of lung volume reduction, a decrease in FRC is associated with improved diaphragm mechanics^28^. Interestingly, the magnitude of FRC reduction observed in the present study was within the range reported after other interventions targeting hyperinflation, e.g. bronchoscopic lung volume reduction^29^. However, these interventions differ fundamentally in mechanism and clinical context and should therefore not be compared directly. These findings raise the possibility that specific patient subgroups may derive greater benefit from feedback-based interventions, analogous to the heterogeneous response observed in lung volume reduction therapies.

The observed decrease in specific airway resistance (sRaw) in the control group, compared with the intervention group needs careful interpretation. As specific airway resistance is defined as the product of airway resistance and lung volume (sRaw = Raw x FRC), one possible interpretation is that a reduction in sRaw with stable FRC suggests decreased airway resistance^30^. Conversely, in the intervention group, reduced FRC may have altered airway mechanics. Reduced lung volumes can decrease radial traction on the airways, potentially leading to relative airway narrowing and thereby attenuating expected reductions in sRaw despite improvements in hyperinflation^30,31^. However, sRaw is known to be highly dependent on breathing pattern and patient cooperation during body plethysmography. In patients with COPD, tidal expiratory flow limitation (tEFL) is common and may affect the accuracy and reproducibility of plethysmographic resistance measurements. As a result, changes in sRaw may reflect a combination of altered breathing mechanics, lung volume changes, and measurement-related factors, rather than isolated changes in airway calibre. Given these physiological and methodological considerations, sRaw should be interpreted cautiously, and may be of limited value as a primary physiological endpoint in this context.

The degree of hyperinflation in COPD is closely linked to symptom burden and functional limitation^27^. Reductions in hyperinflation have been associated with improvements in dyspnoea and overall symptom perception. Accordingly, subjective improvements might have been anticipated. However, in the present study, no significant changes in CAT scores were observed, and subjective assessments showed no consistent trend. A single feedback intervention may be insufficient to induce sustained changes in breathing behaviour. Durable improvements in respiratory mechanics and symptom perception likely require repeated training and reinforcement over time. Most existing inhalation training studies employed repeated sessions to consolidate technique over time. François and colleagues^32^ for example, used graphical breathing feedback over 20–35 sessions, yielding improvements in FEV1 and FVC. Unlike their protocol, our single-session approach may have been insufficient to affect subjective outcomes, suggesting that repeated training with EIT-guided feedback may yield greater clinical benefit.

Nonetheless, to our knowledge, only one other study has explored the role of EIT as a feedback tool. Yang and colleagues^33^ demonstrated that single-session EIT feedback improved breathing technique during pursed-lip breathing and resulted in a more homogeneous ventilation distribution. Importantly, in their study, participants were not instructed to optimize ventilation homogeneity directly. Instead, the visual feedback targeted breathing variables under voluntary control, such as breathing depth, expiratory flow time, and the inspiratory-to-expiratory ratio, whereas ventilation homogeneity was evaluated as an outcome measure. Similarly, in our study, participants were not instructed to optimize specific EIT-derived indices such as TID, GI, RVDI. Rather, they were encouraged to maximize the visible ventilated area during inspiration and maintain a stable image during breath-holding, while TID, GI and RVDI were analysed as exploratory physiological outcome measures. No significant between-group differences were observed. However, RVDI increased in the intervention group from 8.1 to 11.5 indicating more delayed ventilation during inhalation compared with the control group. This may reflect a more conscious inhalation manoeuvre as participants in the intervention group were able to view their ventilation status in real-time. The absence of significant EIT differences does not necessarily contradict effective inhalation because EIT was not designed to quantify peripheral drug deposition. The EIT device used displayed only a two-dimensional cross-sectional image of the lung, with the anatomical regions represented depending on electrode belt position. In our study, the belt was positioned between the 4th–6th intercostal spaces according to the recommendations available when the study protocol was developed. More recent consensus recommendations favour placement at the 4th–5th intercostal spaces. Thus, variation in belt position, particularly when positioned more caudally, may have influenced the derived EIT parameters and should be considered when interpreting the absence of significant between-group differences. A limiting factor that may have affected the statistical power of EIT-derived parameters is the number of patients eligible for analysis. In the intervention group, only 11 patients had data of sufficient quality for inclusion. This limited sample size may have affected not only EIT-derived parameters but also other study endpoints. Larger cohorts will be required in future studies to improve statistical power and allow more robust evaluation of physiological effects. Future studies should therefore examine whether repeated EIT-guided inhalation training can lead to sustained improvements in regional ventilation distribution or clinical outcomes and further examine which EIT features, if any, capture inhalation quality. Although COPD stages were comparable between groups, randomization was not stratified according to disease severity, and a small imbalance in the distribution of GOLD stage I patients occurred by chance. However, the overall distribution of COPD severity did not differ significantly between groups (p = 0.400). Given the limited sample size of this pilot study, baseline imbalances cannot be completely excluded and should be considered when interpreting the findings. In addition, baseline CRP levels differed significantly between groups. However, median CRP levels were low in both groups (< 10 mg/L) making a clinically relevant inflammatory exacerbation unlikely^34^, and all participants were clinically stable without evidence of an acute exacerbation. Future studies may benefit from more balanced inclusion across disease severity and from targeting patients with a pronounced hyperinflation phenotype, who may derive the greatest benefit from visual feedback-guided inhalation strategies. Another limitation concerns the timing of post-bronchodilator lung function testing. According to the study protocol, body plethysmography was performed 5–15 min after inhalation. As the maximal bronchodilator effect of salbutamol may not have been reached in participants assessed at the earlier end of this interval, this may have caused additional variability in the measured lung function responses. However, the same predefined protocol was applied to both randomized groups, making a systematic bias towards either group less likely. Nevertheless, this variability should be considered when interpreting the between-group differences observed in this small pilot study.

## Conclusion

An essential aspect of inhalation therapy for COPD is the precise application and effective deposition of medication, which can be optimized through regular training and education. Implementing a feedback system, such as visual real-time feedback via lung EIT, may further enhance training effectiveness and improve inhalation and breathing mechanics. In this study, a single training session using EIT was associated with a greater reduction in FRC than standard inhalation training, suggesting that real-time visual feedback may help reduce hyperinflation in patients with COPD. These findings should be confirmed in larger, adequately powered studies.

## Supplementary Information

Below is the link to the electronic supplementary material.

Supplementary Material 1

Supplementary Material 2

## Data Availability

Data can be provided on reasonable request addressed to the corresponding author. All data sharing statements are subject to conformity with German data protection legislation and rules (Datenschutzgrundverordnung—DSGVO).

## Declarations

### Use of AI

AI-assisted tools were used (ChatGPT, Version 5.1) only for language editing (grammar and readability). No AI tools were used for study design, data analysis, interpretation, or generation of scientific content. All intellectual contributions and verification of the manuscript were performed by the authors.
