# Supplementary material for: Visual feedback using electrical impedance tomography during inhalation therapy for chronic obstructive lung disease: a pilot study
Source: Sci Rep. 2026 Aug 24;16:26527. doi: 10.1038/s41598-026-67510-0 (PMC13503773; doi:10.1038/s41598-026-67510-0)
Supplement: Supplementary file 1 [file 41598_2026_67510_MOESM2_ESM.docx]

| **Supplementary Table 5: EIT-derived parameters and CAT follow-up assessment** of COPD patients in the control group (n = 15) compared to the intervention group (n = 11). For EIT-derived parameters, baseline values, inhalation values and calculated differences before and during inhalation are shown. Here, TID is the calculated difference between baseline and inhalation. Baseline values were recorded during 3 – 5 resting breathing cycles before inhalation. | | | |
| --- | --- | --- | --- |
|  | **Control; N = 15**  **median (Q1 – Q3)** | **Intervention; N = 11**  **median (Q1 – Q3)** | **p-value** |
| TID | 237 (136 - 348) | 277 (136 - 348) | .921 |
| GI baseline | 48.9 (45.6 – 56.5) | 53.9 (44.7 – 43.2) | .166 |
| GI inhalation | 42.8 (39.4 – 49.9) | 45.3 (41.4 – 49.7) | .088 |
| GI difference | -4.02 (-7.3 – 0.04) | -8.3 (-20 – -1.4) | .242 |
| RVDI baseline | 7.4 (6.7 – 8.1) | 8.1 (7.1 – 8.9) | .259 |
| RVDI inhalation | 7.6 (4.5 – 12.3) | 11.5 (8.3 – 23.3) | .071 |
| RVDI difference | 0.2 (-0.9 – 4.1) | -0.2 (-1.1 – 16.4) | .101 |
| CAT score before | 26 (8 – 37) | 24.5 (8 – 36) | .949 |
| CAT score after | 23 (6 – 31) | 27.5 (4 – 35) | .176 |
| CAT score diff. | -2.4 (-9 – 6) | -1 (-10 – 6) | .631 |
| **TID:** Tidal Impedance Difference before and during inhalation; **GI:** Global Inhomogeneity Index; **RVD [SD]:** Regional Ventilation Delay [Standard Variation]; **CAT:** COPD assessment test; | | | |

| **Supplementary Table 6: Quantitative evaluation of visual biofeedback** in the intervention group (n = 12). | |
| --- | --- |
|  | **Intervention; N = 12**  **median (Q1 – Q3)** |
| Did the EIT image provide any benefit to you? | 4 (1 – 5) |
| Did you understand the EIT image? | 4.5 (3 – 5) |
| Has he study given you a better sense of how well you are inhaling? | 4 (1 – 5) |
| Was the guidance and feedback from us helpful to you? | 5 (3 – 5) |
| Has the effect of your inhalation medication improved (e.g., through deeper inhalation, etc.)? | 3 (1 – 4) |
| **Rating scale:** (5) = strongly agree; (4) = agree; (3) = neutral / neither agree nor disagree; (2) = disagree; (1) = strongly disagree; | |
